# Expanding the Phenotypic Spectrum Associated with DPH5-Related Diphthamide Deficiency

**DOI:** 10.3390/genes16070799

**Published:** 2025-07-02

**Authors:** Davide Politano, Cecilia Mancini, Massimiliano Celario, Francesca Clementina Radio, Fulvio D’Abrusco, Jessica Garau, Silvia Kalantari, Gaia Visani, Simone Carbonera, Simone Gana, Marco Ferilli, Luigi Chiriatti, Camilla Cappelletti, Katia Ellena, Elena Prodi, Renato Borgatti, Enza Maria Valente, Simona Orcesi, Marco Tartaglia, Fabio Sirchia

**Affiliations:** 1 Department of Brain and Behavioural Sciences, University of Pavia, 27100 Pavia, Italy; davide.politano01@universitadipavia.it (D.P.); renato.borgatti@mondino.it (R.B.); simona.orcesi@mondino.it (S.O.); 2 Child Neurology and Psychiatry Unit, IRCCS Mondino Foundation, Via Mondino 2, 27100 Pavia, Italy; 3 Molecular Genetics and Functional Genomics, Bambino Gesù Children’s Hospital, IRCCS, 00146 Rome, Italy; cecilia.mancini@opbg.net (C.M.); francesca.clementina.radio@gmail.com (F.C.R.); marco.ferilli@opbg.net (M.F.); luigi.chiriatti@opbg.net (L.C.); camilla.cappelletti@opbg.net (C.C.); marco.tartaglia@opbg.net (M.T.); 4 Neurogenetics Research Center, IRCCS Mondino Foundation, 27100 Pavia, Italy; fulvio.dabrusco01@universitadipavia.it (F.D.); jessica.garau@mondino.it (J.G.); simone.gana@mondino.it (S.G.); enzamaria.valente@unipv.it (E.M.V.); 5 Department of Molecular Medicine, University of Pavia, 27100 Pavia, Italy; silvia.kalantari@unipv.it (S.K.); gaia.visani01@universitadipavia.it (G.V.); simone.carbonera01@universitadipavia.it (S.C.); fabio.sirchia@unipv.it (F.S.); 6 Medical Genetics Unit, IRCCS San Matteo Foundation, 27100 Pavia, Italy; 7 Department of Biomedicine and Prevention, University “Tor Vergata”, 00173 Rome, Italy; 8 Department of Clinical, Surgical, Diagnostic and Pediatric Sciences, University of Pavia, 27100 Pavia, Italy; katia.ellena01@universitadipavia.it; 9 Neuroradiology Department, IRCCS Mondino Foundation, 27100 Pavia, Italy; elena.prodi@mondino.it

**Keywords:** DPH5, diphtamide deficiency, neurodevelopmental disorder, ribosomopathy

## Abstract

Background/Objectives: Neurodevelopmental disorders (NDDs) represent a clinically diverse group of conditions that affect brain development, often leading to varying degrees of functional impairment. Many NDDs, particularly syndromic forms, are caused by genetic mutations affecting critical cellular pathways. Ribosomopathies, a subgroup of NDDs, are linked to defects in ribosomal function, including those involving the synthesis of diphthamide, a post-translational modification of translation elongation factor 2 (eEF2). Loss-of-function (LoF) mutations in genes involved in diphthamide biosynthesis, such as *DPH1*, *DPH2*, and *DPH5*, result in developmental delay (DD), intellectual disability (ID), and multisystemic abnormalities. DPH5-related diphthamide deficiency syndrome has recently been reported as an ultrarare disorder linked to LoF mutations in *DPH5*, encoding a methyltransferase required for diphthamide synthesis. Methods: Clinical, neurological, and dysmorphological evaluations were performed by a multidisciplinary team. Brain MRI was acquired on a 3T scanner. Craniofacial abnormalities were assessed using the GestaltMatcher phenotyping tool. Whole exome sequencing (WES) was conducted on leukocyte-derived DNA with a trio-based approach. Bioinformatic analyses included variant annotation, filtering, and pathogenicity prediction using established databases and tools. Results: The affected subject carried a previously reported missense change, p.His260Arg, suggesting the occurrence of genotype–phenotype correlations and a hypomorphic behavior of the variant, likely explaining the overall milder phenotype compared to the previously reported patients with *DPH5*-related diphthamide deficiency syndrome. Conclusions: Overall, the co-occurrence of short stature, relative macrocephaly, congenital heart defects, variable DD/ID, minor skeletal and ectodermal features, and consistent craniofacial features suggests a differential diagnosis with Noonan syndrome and related phenotypes.

## 1. Introduction

Neurodevelopmental disorders (NDDs) are a heterogeneous group of conditions affecting brain development and impairing the autonomy of affected individuals with variable severities [1]. NDDs may occur in the frame of complex syndromic entities characterized by multisystem involvement with organ malformations and/or extra-neurological functional alterations [2,3]. Up to 40% of isolated intellectual disability (ID) and syndromic NDDs have an established underlying monogenic defect, primarily due to de novo gene variants [4], and implicating various cellular pathways that are critical for brain development and function [5]. Among these disorders, ribosomopathies represent a relatively large group of clinically diverse and genetically heterogeneous diseases associated with altered ribosomal function due to defects involving ribosomal proteins, rRNA processing, or ribosome assembly [6,7].

Among ribosomopathies, deficiency in diphthamidation of eukaryotic translation elongation factor 2 (EEF2) due to biallelic LoF variants in genes encoding diphthamide synthesis enzymes is causally related to a phenotypic spectrum characterized by developmental delay (DD)/ID, short stature, craniofacial dysmorphisms, and ectodermal anomalies [8]. Diphthamide is a post-translationally modified histidine residue that is found in eukaryotic and archaeal translation elongation factor 2 (EF2), a protein with a crucial function in ribosome-dependent protein synthesis [9,10]. The biosynthesis of diphthamide is complex, involving multiple enzymes that are collectively known as diphthamide biosynthesis proteins (DPH), namely from DPH1 to DPH7, and the methylating cofactor S-adenosylmethionine (SAM) [11]. Alterations in the diphthamide biosynthesis pathway, specifically in DPH1 or DPH2 activity, have been associated with rare syndromic NDDs in humans [12,13,14].

In 2022, Shankar and colleagues provided compelling evidence causally linking LoF variants in the DPH5 gene, which encodes a key component of diphthamide synthesis, to a previously unrecognized NDD [15]. They described five patients from three unrelated families who shared a complex phenotype characterized by DD/ID, multisystemic abnormalities, and peculiar craniofacial dysmorphisms. Each family carried a unique set of biallelic variants in the gene, which were functionally validated to disrupt protein function.

**Figure 1:**
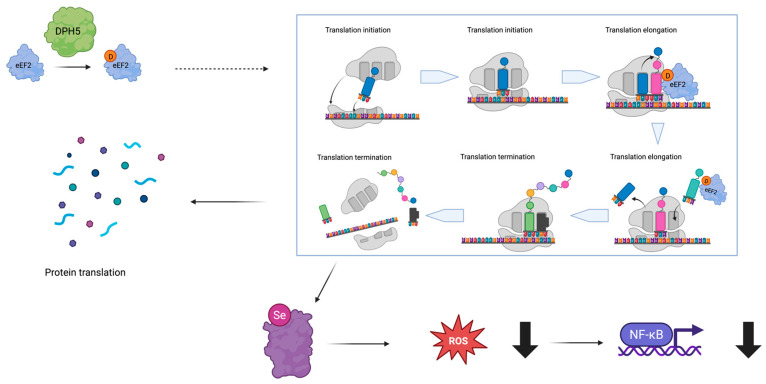
DPH5 and the diphthamidation pathway. DPH5 is important in diphthamide synthesis, which is important in the regulation of protein translation in general, particularly in selenoprotein translation and hence oxidative stress balance, although other pathways may be feasible but are not known to date. In the top left, DPH5 catalyzes the diphthamidation of eEF2 (black arrow denotes the conversion of non-diphthamidated to diphthamidated eEF2 through DPH5 activity). In the right panel, diphthamidated eEF2 facilitates efficient protein translation (indicated by grey arrows representing sequential translation steps; grey components depict the ribosome; colored rectangles represent tRNAs carrying amino acids shown as colored circles; the colored line along which ribosomes and tRNAs assemble represents mature mRNA with its nucleotide sequence), visualized in the bottom left (connected via a black arrow from right to left). It also promotes selenoprotein synthesis (indicated by a black arrow from top right to bottom left), shown in the bottom center. Selenoproteins contribute to the reduction (thick downward arrow) of reactive oxygen species (ROS, indicated by a black arrow), ultimately leading to decreased activation of the NF-κB signaling pathway. Created in BioRender (https://www.biorender.com/).

*DPH5*, located on chromosome 1p21.2, is a highly conserved gene among eukaryotes. It is composed of eight exons and encodes different isoforms, which are the result of alternative transcript processing. DPH5 is a ubiquitously expressed protein that is part of a complex pathway required to synthesize diphthamide, a SAM-derived unique post-translational modification only found in archaeal and eukaryotic EF2 [9,10]. Diphthamide is specifically important in the regulation of selenoprotein translation and hence oxidative stress balance; thus, reduction in diphthamidation leads to NF-kb pathway hyperactivation (Figure 1) [16]. The NF-kb pathway is a very important and pleiotropic pathway in neuronal and non-neuronal cell development [17], strengthening the link between DPH5 activity alteration and syndromic NDDs. Diphthamide modification is dependent upon three complex series of reactions: the first biosynthetic step is carried out by the DPH1–DPH2 heterodimer that forms a 3-amino-3-carboxypropyl (ACP)-modified histidine upon electron donation by DPH3 and J-type chaperone DPH4; the second step leads to the generation of methylated diphthine from the ACP-modified intermediate by DPH5, followed by targeted demethylation reactions of conserved residues catalyzed by DPH7; the last step is the amidation reaction to generate fully diphthamide-modified eEF2 by DPH6 [11]. Consistently, LoF biallelic variants in three members of the above-mentioned associated pathway, specifically DPH1 [12], DPH2 [13,14], and DPH5 [15], have been associated with severe syndromic NDDs.

Here, we present the case of a 10-year-old boy affected by a multisystemic condition and carrying a homozygous pathogenic missense variant in *DPH5*, providing data that further expand the phenotypic spectrum associated with DPH5-related diphthamide syndrome.

## 2. Materials and Methods

Clinical, neurological, and dysmorphological evaluation was performed by a multidisciplinary team of clinical geneticists (F.S., S.G.), neuropsychiatrists (S.O., D.P., M.C.), and neuroradiologists (K.E., E.P.). Following signed informed consent from the patient’s parents, clinical data were acquired from the medical records in IRCCS Mondino Foundation (Pavia, Italy) and enrolled in the research project dedicated to undiagnosed patients, within the framework of Rete Italiana Salute dell’Età Evolutiva (Rete IDEA). The proband underwent a brain MRI 3T under sedation with acquisition of multiplanar T1- and T2-weighted images with age-appropriate TR and TE values.

To ameliorate the assessment of the craniofacial abnormalities, a computer-aided next-generation phenotyping tool named GestaltMatcher (University of Bonn, Bonn, Germany) was used [18]. GestaltMatcher is an extension of DeepGestalt and quantifies the facial syndromic similarity between two patients. It encodes each individual picture into a 320-dimensional facial phenotype descriptor (i.e., Clinical Face Phenotype Space—CFPS). A cosine distance was calculated in the CFPS to objectify the facial syndromic similarity between two pictures. A small distance between two pictures correlates to a high facial syndromic similarity. The pictures were ranked in ascending order based on the cosine distance. The facial phenotype of our patient was compared with the GestaltMatcher gallery.

Whole exome sequencing (WES) was performed on leukocyte-derived DNA samples using a trio-based approach. The SureSelect QXT Human All Exon V7 kit (Agilent, Santa Clara, CA, USA) was used for target region enrichment, and sequencing was conducted on an Illumina NovaSeq6000 platform (Illumina, San Diego, CA, USA). The raw data were processed using an in-house pipeline, as previously described [19,20], in line with the GATK’s Best Practices [21]. The UCSC GRCh37/hg19 genome assembly was used as a reference for read alignment, using the BWA-MEM tool [21,22], and variant calling was carried out using HaplotypeCaller (GATK v3.7, Broad Institute, Cambridge, MA, USA). SnpEff v.5.0 and dbNSFP v.4.2 tools were used for variants’ functional annotation [23]. Public (dbSNP150 and gnomAD V.2.0.1) and in-house (approximately 3300 exomes) databases were used to filter out variants with MAF higher than 0.001. Combined Annotation Dependent Depletion (CADD) v.1.64 [24], Mendelian Clinically Applicable Pathogenicity (M-CAP) v.1.0 [25], and Intervar v.2.0.1 [26] were considered for functional impact prediction. WES data output is summarized in Supplemental Table S1.

Sanger sequencing was carried out for confirmation of variants and segregation.

## 3. Results

### 3.1. Clinical Assessment

**Figure 2:**
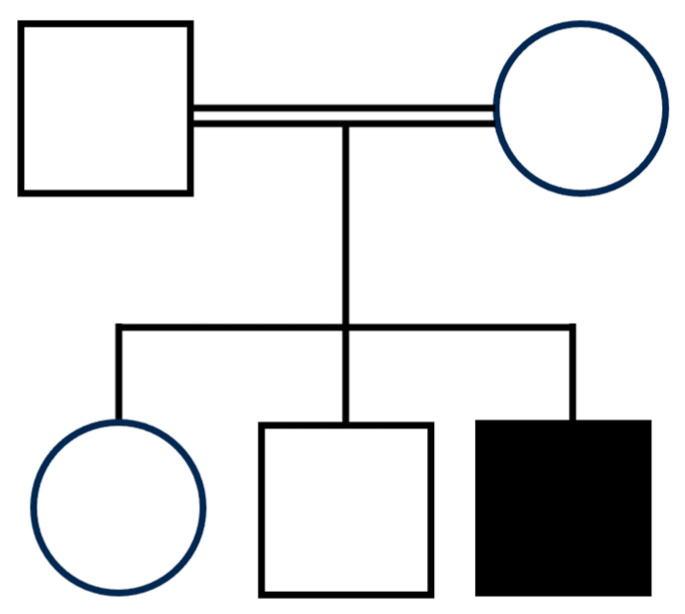
Family tree. The top white square and circle represent the consanguineous parents of the proband (father and mother, respectively). The bottom white circle and square represent healthy siblings of the proband (sister and brother, respectively), and the black square represents the described patient.

The proband was a 10-year-old boy, the third child born to non-consanguineous parents of Egyptian origin (Figure 2). Family history was negative for neuropsychiatric disorders. Pregnancy was regular, delivery occurred by cesarean section due to a previous cesarean section, and the perinatal course was depicted as normal except for moderate feeding problems that were characterized by chewing and swallowing difficulties. Motor development was severely delayed, with head and trunk control achieved at three years of age and independent, but unstable, walking at six years. Expressive language was absent. The patient experienced maintenance insomnia with frequent awakenings and episodes of apnea during sleep, starting in the first year of life. From 18 months of age, rare episodes of cyanosis, eye deviation, loss of consciousness, and hypertonia were observed, initially in the absence of a clear electroencephalographic consistency. At the age of seven, the frequency of these episodes increased, which were accompanied by electroencephalogram (EEG) abnormalities, including poor organization, and interhemispheric asymmetry with slower activity on the left hemisphere that was characterized by theta–delta rhythmic activity on left temporo-occipital region with spike and spike-and-wave epileptic anomalies that diffused contralaterally and worsened during sleep, in the absence of typical NREM sleep EEG features. Based on findings, pharmacological treatment was initiated, initially with valproic acid and clobazam, and later with valproic acid and levetiracetam, resulting in a relatively good seizure control.

**Figure 3:**
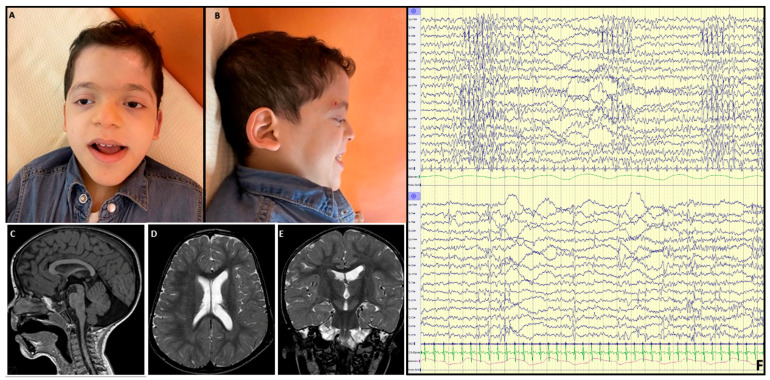
Craniofacial dysmorphism and brain imaging: (**A**,**B**) frontal, and side view of proband’s craniofacial dysmorphisms (i.e., coarse facial features, bitemporal narrowing, bilateral frontal and parietal bossing, high forehead, triangular face, synophrys, epicanthus, hypertelorism, bilateral ptosis, depressed nasal bridge, low-set overfolded dysmorphic ears with thick earlobes, long deep philtrum, thick lips, pointed chin). (**C**–**E**) A brain MRI was performed at the age of 3. (**C**) sagittal T1-weighted MRI image shows mild hypoplasia of posterior corpus callosum and mild inferior vermis hypoplasia with enlarged cisterna magna; (**D**) and (**E**) respectively, axial and coronal T2w MRI images showing lateral ventricles asymmetry with few dilated perivascular spaces (**D**) and left incomplete hippocampal inversion (**E**). (**F**) EEG shows poor organization with multifocal spike and wave discharges, prevalent in temporo-posterior regions.

At last evaluation (11 years and 4 months), the neurological and neuropsychiatric phenotype was characterized by profound ID with a happy disposition, absence of language, neuro-ophthalmologic abnormalities characterized by nystagmus, diffuse hypotonia, joint laxity, global muscle hypotrophy, pharmacologically responsive generalized epilepsy, and absent sphincter control. Craniofacial dysmorphisms were noted, including plagiocephaly, bilateral frontal and parietal bossing, features suggestive of a RASopathy (i.e., hypertelorism, bilateral mild ptosis, epicanthus, blue sclerae, coarse facial features, low set ears, depressed nasal bridge, and pointed chin), kyphosis, and pes planus (Figure 3 and Table 1). Multisystemic involvement was observed, including short stature, which was treated with growth hormone (GH) replacement therapy, frequent respiratory infections leading to multiple hospitalizations, moderate obstructive sleep apnea, and gastroesophageal reflux disease.

EEG documented a poor organization with multifocal spike and wave discharges, prevalent in temporo-posterior regions; polysomnography revealed moderate obstructive sleep apnea; brainstem auditory evoked potentials (BAEPs) analysis demonstrated a reproducible peak I, while peak III was not reproducible, and peak V was poorly reproducible. Brain magnetic resonance imaging (MRI) at the age of 3 showed callosal hypoplasia, multiple dilated perivascular spaces, a smaller left hippocampus with globular appearance, and mild inferior vermis hypoplasia associated with an enlarged cisterna magna (Figure 3C–F). Other instrumental examinations, including chest computed tomography, echocardiogram, and electrocardiogram–Holter, metabolic screening comprehensive of plasma and urinary amino acids and organic acids, and abdominal ultrasound, provided results within the normal limits.

### 3.2. Computational Facial Analysis

Due to the clinical impression of facial features suggestive of a RASopathy, we used the GestaltMatcher tool to objectify this finding [18]. Characteristic facial features were confirmed in the averaged facial analysis, showing YWHAZ-related disorder [27], Noonan syndrome (OMIM#163950) and cardiofaciocutaneous syndrome (CFCS, OMIM#115150) in the top 10 list with a distance of 0.657, 0.686, and 0.692, respectively. The same analysis was performed on subject 1B by Shankar et al. [15], who were homozygous for the same missense variant, returning Noonan syndrome and CFCS in the top 10 list with a distance of 0.631 and 0.702, respectively. While subjects 2A, 2B, and 3 show Noonan syndrome-like disorder with loose anagen hair (OMIM#607721), they show a distance of 0.733 and CFCS (distance 2B: 0.746 and 3:0.764) as the most similar RASopathies.

### 3.3. Molecular Analyses

Given the patient’s complex clinical presentation, genetic etiology was suspected, prompting genetic testing. Chromosomal microarray analysis revealed no pathogenic duplications or deletions. Trio-based WES analysis allowed for the identification of a homozygous missense variant (c.779A > G; p.His260Arg; NM_015958.3) in *DPH5*. This variant, previously reported in two siblings by Shankar et al. [15] and classified as pathogenic according to ACMG criteria (PM2, PS4, PS3, PP1, PP5), was found in gnomAD with a frequency of 0.000008. Segregation analysis confirmed the heterozygous state for the pathogenic variant in both parents.

## 4. Discussion

DPH5-related diphthamide deficiency syndrome is a recently identified and still poorly defined genetic disorder, with only five patients reported in the literature to date [15]. It is characterized by severe clinical manifestations, ranging from neonatal death to profound neurodevelopmental impairment, accompanied by craniofacial dysmorphisms, neurological and extra-neurological dysfunctions, and various malformations (see Table 1). It is included among ribosomopathies [21].

Ribosomopathies are genetic disorders caused by mutations in ribosomal constituents or in factors with a role in ribosome assembly, disrupting ribosome function and leading to tissue-specific clinical manifestations despite the universal need for ribosomes. Well-known examples include Diamond–Blackfan anemia, Shwachman–Diamond syndrome, dyskeratosis congenita, cartilage-hair hypoplasia, and Treacher Collins syndrome. The most prominent features include bone marrow failure, resulting in anemia and low blood cell counts, often diagnosed in early childhood, alongside developmental abnormalities such as growth retardation, craniofacial malformations, and limb defects. Some patients also display immune deficiencies, skin, nail, or hair changes, pancreatic or liver dysfunction, and NDDs. Notably, these conditions increase the risk of cancer, especially hematologic malignancies, with the risk more elevated for specific cancers. The disease course is often paradoxical, transitioning from hypo-proliferation (e.g., anemia) in early life to hyper-proliferation and cancer risk later—Dameshek’s riddle—with variable presentation and severity [28].

We herein describe the sixth patient affected by DPH5-related syndrome and the second family carrying the p.His260Arg variant. Our patient presented with a history of severe global DD, profound ID, nearly absent expressive language, nystagmus, craniofacial and body dysmorphisms, gastrointestinal issues, and growth retardation, as previously described in all the previously reported patients [15]. Additionally, he showed epilepsy, which had previously been reported in a subset of the originally identified affected individuals, as well as less common features, including sleep difficulties (40%), obstructive sleep apnea (40%), and frequent respiratory infections (40%). EEG findings were severely abnormal, and brain MRI showed marked, although non-specific, abnormalities across multiple brain regions consistent with the majority of patients previously reported [15]. Notably, BAEPs showed abnormalities in the reproducibility of waves originating from pontine structures, a novel finding not previously associated with this syndrome. Cardiac anomalies were not observed in our patient, although described in all the patients reported in the literature to date (see Table 1).

Furthermore, two additional patients (ClinVar accessions SCV003841335.1 and SCV004238160.1) carrying the same variant identified in our patient have been reported in ClinVar. The individual corresponding to SCV003841335.1 presented with seizures, hypogammaglobulinemia, malnutrition, and oropharyngeal dysphagia; no phenotypic information was provided for SCV004238160.1 (Variant information was retrieved from ClinVar, https://www.ncbi.nlm.nih.gov/clinvar/, accessed on 25 June 2025).

The co-occurrence of short stature, which was responsive to GH supplementation therapy, relative macrocephaly, variable congenital heart defects, variable DD/ID, minor skeletal and ectodermal features, and recognizable craniofacial features (i.e., coarse facial features, bitemporal narrowing, bilateral frontal and parietal bossing, high forehead, epicanthal folds, hypertelorism, bilateral ptosis, deep-set eyes, broad and depressed nasal bridge, low-set ears, overfolded dysmorphic ears with thick earlobes, long deep philtrum, downturned corners of mouth, thick lips, pointed chin) suggests a differential diagnosis with Noonan syndrome and related conditions, as supported by GestaltMatcher analysis. Notably, these diseases, which are collectively termed RASopathies, are caused by germline pathogenic variants in genes that encode components and regulators of the RAS-mitogen-activated protein kinase (MAPK) signaling pathway [29,30,31]. DD/ID and speech delay/learning difficulties seem to be more severe in the DPH5-related syndrome, with seizures reported in a subset of individuals. Although a strict interaction between DPH5 and the RAS-MAPK pathway is not apparent, indicating the DPH5-related condition as a RASopathy “phenocopy”, Dph5 knockdown was demonstrated to ameliorate the pathogenic phenotypes in an in vitro model of Ras-induced tumor [32]. Moreover, the impact of activating mutations affecting the RAS-MAPK circuits results in NF-kb constitutive activation in several types of tumors [33,34], as expected in the dysregulation of selenoprotein translation [17] induced by DPH5-related diphthamide deficiency.

Shankar and colleagues generated a *Dph5*^His260Arg^ mouse model with a highly lethal phenotype in homozygous mice and a variably penetrant multisystemic disorder in heterozygote mice. The latter phenotype resembled the human biallelic phenotype, possibly due to the inbreeding and consequent susceptibility to other homozygous uncharacterized variants. They also confirmed, through in vitro phenotype assays using *Saccharomices cerevisiae* as a model system, that diphthamide synthesis was only partially compromised in strains expressing dph5His260Arg compared to the strains carrying nonsense and frameshift dph5 variants. Indeed, the latter types of variants determined a complete or severely defective protein function. In silico structural models performed to explore the consequences of the His260Arg substitution, although the residue being highly conserved accross species, did not document substantial conformational rearrangements of the protein or misfolding but showed a possible perturbation of the solvent-exposed surface of the protein binding to eEF2, predicting a reduced interaction between the two proteins [15].

Notwithstanding the limited available functional characterization of the variant, based on the relatively milder phenotype associated with the His260Arg substitution, we anticipate a likely hypomorphic behavior of this missense variant.

## 5. Conclusions

In conclusion, our case strengthens and broadens the clinical and radiological findings in DPH5-related syndrome and identifies His260 as a mutational hotspot associated with an overall milder phenotype, possibly resulting from residual DPH5 function.

## Figures and Tables

**Table 1:** Molecular and phenotypic characteristics of individuals with *DPH5* variants. Comparing our patient to those described by Shankar et al. [15].

Features	DPH5 Variant and Inheritance Model	Age	Sex	Positive Family History	Weight (SD If p < 3rd p)	Height (SD If p < 3rd p)	Head Circumference (SD if p < 3rd p)	Facial Features		Prenatal/Birth History	Perinatal History	Global Developmental Delay	Independent Ambulation	Language
Present case	Hz c.779A>G (p.His260Arg)	10 y	M	No	3rd p (−2 SD)	<3rd p (−3.7 SD)	50th–75th p	Coarse facial features, bitemporal narrowing, bilateral frontal and parietal bossing, high forehead, triangular face, synophrys, epicanthus, hypertelorism, bilateral ptosis, blue sclerae, depressed nasal bridge, low-set ears, overfolded dysmorphic ears with thick ear lobe, long deep philtrum, thick lips, pointed chin.		Normal pregnancy, Cesarean section for previous cesarean section.	Feeding difficulties	Yes	Yes, milestone reached at 6 years of age	Non verbal
Patient 1 [15]	Hz c.779A>G (p.His260Arg)	10 y	F	One previous live birth with mortality at 1 week of age. Similarly affected sibling (patient 2).	Failure to thrive, 10th p with G tube feeds.	<3rd p (−2.5 SD)	14th p	Broad forehead, bitemporal narrowing, sparse eyebrows, epicanthal folds, deep-set eyes, broad nasal bridge, rounded tip of nose, downturned corners of mouth, high arched palate, triangular chin, dental caries.		Decreased fetal movement in pregnancy, born at 42 weeks.	Neonatal intensive care unit stay for hypoxia	Yes	Yes, but limited	Non verbal
Patient 2 [15]	Hz c.779A>G (p.His260Arg)	8 y	M	One previous live birth with mortality at age 1 week. Similarly affected sibling (patient 1).	70th p	10th–25th p	23rd p	Broad forehead, bitemporal narrowing, sparse eyebrows, epicanthal folds, wide palpebral fissures, broad nasal bridge, rounded nasal tip, downturned corners of mouth, triangular chin, multiple dental caries.		Normal pregnancy, normal spontaneous vaginal delivery.	No issues	Yes	No	Non verbal
Patient 3 [15]	Compound het c.619C>T (p.Arg207 *) and c.329A>G (p.Asn110Ser)	9 y	M	Similarly affected sibling (patient 4).	<3rd p (−5SD)	<3rd p (−4.99 SD)	13th p	High forehead, high anterior hairline, depressed midface, upslanting eyes, sparse eyelashes, mild epicanthal folds, thick alveolar ridges.		Decreased fetal movement, excess fluid at delivery.	Hospital stay for 3 weeks for poor feeding	Yes	No	Non verbal
Patient 4 [15]	Compound het c.619C>T (p.Arg207 *) and c.329A>G (p.Asn110Ser)	1 y	F	Similarly affected sibling (patient 3).	<3rd p (−3.45 SD)	<3rd p (−3.06 SD)	32nd p	Prominent forehead, depressed midface, broad alveolar ridges, faint eyebrows, upslanting eyes.		Excess fluid at delivery.	No issues	Yes	NA for age	Non verbal
Patient 5 [15]	Hz c.521dupA (p.Asn174LysfTer10)	11 d	F	Spontaneous miscarriages, one antepartum stillbirth, and one neonatal death. Microphthalmia, hypertelorism, IUGR, TOF, hydrocephalus, and cerebellar hypoplasia in dead babies.	BW 800 gm	NA	NA	Broad forehead, bitemporal narrowing, sparse eyebrows, epicanthal folds, wide palpebral fissures, deep-set eyes, broad nasal bridge, rounded nasal tip, downturned corners of mouth, triangular chin, micrognathia.		Polyhydramnios. Preterm baby delivered via Cesarean section for breech presentation	Cyanosis	NA	NA for age	NA
**Features**	**Intellectual disability**	**Behavioral concerns**	**Nervous system dysfunction**	**Brain imaging studies (age)**	**EEG**	**Hearing**	**Cardiac system**	**Respiratory system**	**Gastrointestinal** **system**	**Endocrinology**	**Musculoskeletal**	**Urogenital**	**Ophthalmology**	**Dermatology**
Present case	Profound	Happy disposition	Seizures, Diffuse hypotonia, sleep difficulties	Brain MRI (3 y): corpus callosum hypoplasia, inferior vermis hypoplasia, enlarged cisterna magna.	Poor organization with multifocal spike and wave discharges, prevalent in the temporo-posterior regions.	Slightly altered BAEP	Normal	Recurrent episodes of cyanosis and apnoea. Frequent infections and moderate obstructive sleep apnea.	GERD	Short stature (started GH replacement therapy)	Kyphosis, joint laxity, hypotonia, and global hypotrophy, pes planus.	Incontinence	Strabismus, abnormal ocular movements (nystagmus)	Normal
Patient 1 [15]	Profound	Screaming episodes	Congenital hypotonia, cerebral palsy, sleep difficulties	Brain MRI (1 y): brain atrophy. CT scan (8 y): within normal limits.	Abnormal, continuous slowing consistent with mild to moderate encephalopathy.	Normal BAEP	Trivial mitral prolapse and regurgitation, normal ECG	Normal	Feeding difficulties, dysphagia, GJ-tube fed, bowel incontinence	Short stature	Lean muscle mass, small hands and feet, tapered fingers, brachydactyly of the third toe, bilateral.	Urinary incontinence	Fixes and follows	Normal
Patient 2 [15]	Profound	Happy disposition	Seizures, spastic quadriplegia, cerebral palsy, contractures, weakness, DTRs 3+, plantars upgoing	Brain MRI (5 y): within normal limits.	Abnormal, consistent with generalized disturbance of cerebral function with multifocal epileptogenic brain abnormality.	Normal BAEP	Trivial mitral prolapse and regurgitation, sinus tachycardia at ECG	Dyspnea, obstructive sleep apnea, asthma, recurrent aspiration, pneumonia, and oxygen therapy at night.	GJ-tube dependent, hematemesis	Short stature	Lean muscle mass, tapered fingers, pes planus, brachydactyly of 4 toes other than the big toe, bilateral.	Urinary incontinence	Ocular melanocytosis, pathologic high myopia-11 DS, fixes and follows	Normal
Patient 3 [15]	Profound	NA	Seizure, appendicular spasticity, central hypotonia, motor regression	Brain MRI (NA): Focal lesion in white matter of left inferior frontal gyrus, vertically oriented hippocampi, prominence of ventricular system.	Abnormal.	Normal, BAEP NA	Congenital aortic dilatation	Breath-holding spells.	G tube	Short stature	Rocker bottom feet, hyperextensible joints, tapered fingers, right single crease, small hands, and feet.	No known issues	Gray sclera with ocular melanocytosis, strabismus requiring surgery	Extensible skin
Patient 4 [15]	NA for age	NA	Myoclonic seizures, central hypotonia, and appendicular hypertonia	Brain MRI (NA): Diffuse paucity of white matter, cerebellar vermian hypoplasia.	Abnormal, consistent with epileptic myoclonus.	Normal, BAEP NA	Aortic dilatation	Normal	GERD	Short stature	Tapered fingers.	No known issues	Gray sclera, cortical visual impairment	Extensible skin, pale
Patient 5 [15]	NA for age	NA	Congenital hypotonia	Prenatal ultrasound: “Strawberry head”. Brain MRI (NA): bilateral minimal tentorial subdural hemorrhage and enlarged cisterna magna.	NA	NA	Multiple muscular VSD and ASD, hypoplastic pulmonary artery, pericardial effusion	Normal	Bowel perforation at 10 days of life, died the following day.	NA	NA	NA	NA	NA

y, years; d, days; M, male; F, female; Hz, homozygous; Het, heterozygous; IUGR, intra uterine growth restriction;; TOF, tetralogy of Fallot; p, percentile; SD, standard deviation; BW, birth weight; NA, not ascertained; DTR, deep tendon reflexes; BAEP, brainstem auditory evoked potentials; ECG, electrocardiogram; ASD, atrial septal defect; VSD, ventricular septal defect; GERD, gastroesophageal reflux disease; GJ tube, gastrojejunostomy tube; G tube, gastrostomy tube; DS, dioptre sphere; *, nomenclature for stop variant.

## Data Availability

The original contributions presented in this study are included in the article/Supplementary Materials. Further inquiries can be directed to the corresponding author(s).

## Supplementary Materials

The following supporting information can be downloaded at: https://www.mdpi.com/article/10.3390/genes16070799/s1, Table S1: WES statistics and data output.
