# Supplementary material for: Expanding the Phenotypic Spectrum Associated with DPH5-Related Diphthamide Deficiency
Source: Genes (Basel). 2025 Jul 2;16(7):799. doi: 10.3390/genes16070799 (PMC12294659; doi:10.3390/genes16070799)
Supplement: Supplementary file 1 [file genes-16-00799-s001.zip › genes-3708608-supplementary.pdf]

**Supplemental Table S1.** WES statistics and data output.

| | |
|---|---|
| WES enrichment kit | SureSelect QXT Human All Exon V7 kit (Agilent) |
| Sequencing platform | NovaSeq6000 (Illumina) |
| Target regions coverage >2x | 96,8% |
| Target regions coverage >10x | 95,8% |
| Target regions coverage >20x | 94,4% |
| Average depth on target | 136x |
| Total number of rare high-quality variants | 4194 |
| Variants with effect on CDS or affecting splice sites[1] | 4028 |
| Private, clinically associated and low frequency variants[2] | 103 |
| Filtered candidate genes | *DPH5*[3] |

[1]High-quality non-synonymous single nucleotide variants plus indels within coding exons and splice regions (-/+8).

[2]High-quality, functionally relevant variants and either unknown, private or low frequency variants (gnomAD MAF<0.1% and frequency<1% within our exomes database).

[3] *DPH5*: CHR1(GRCh37) c.779A>G, p.His260Arg, CADD: score 24,5
